# Supplementary material for: Human Wnt/β-Catenin Regulates Alloimmune Signaling during Allogeneic Transplantation
Source: Cancers (Basel). 2021 Jul 28;13(15):3798. doi: 10.3390/cancers13153798 (PMC8345079; doi:10.3390/cancers13153798)
Supplement: Supplementary file 1 [file cancers-13-03798-s001.zip › cancers-1281631-supplementary[1]MK.pdf]

Human Wnt/β-catenin Regulates Alloimmune Signaling during Allogeneic Transplantation

Mahinbanu Mammadli, Rebecca Harris, Sara Mahmudlu, Anjali Verma, Adriana May, Rohan Dhawan, Adam T. Waickman, Jyoti Misra Sen, Avery August and Mobin Karimi.

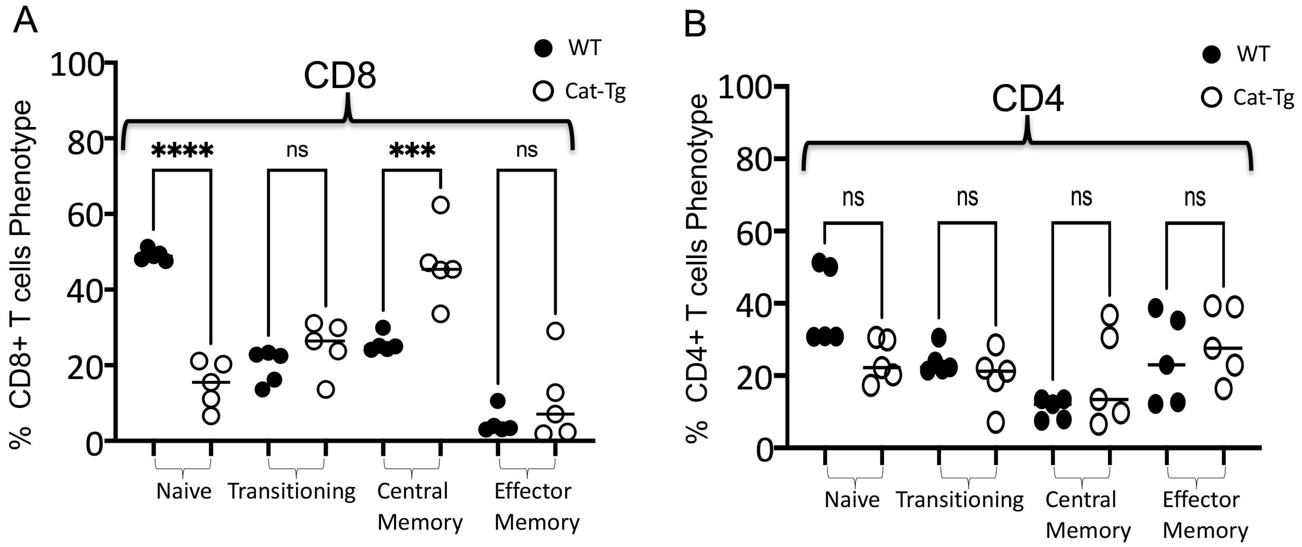

Figure S1. (Related to Figure 2). T cells from Cat-Tg mice exhibit enhanced T cell IMP phenotypes (A–B) Quantitative analysis of T cells from WT and *Cat-Tg* mice that were examined for effector memory, central memory, transitioning/activating, and naïve population frequencies. CD4 (**A**) and CD8 (**B**) T cells were examined for these populations by flow cytometry. Statistical analysis was performed using two-way ANOVA, one-way ANOVA confirmed by Student's *t*-test, p-values are presented. Symbol meanings for P-values are: ns - $p > 0.05$= * $p \leq 0.05$= ** $p \leq 0.01$ *** $p \leq 0.001$= **** $p \leq 0.0001$(n = 5 mice per group).

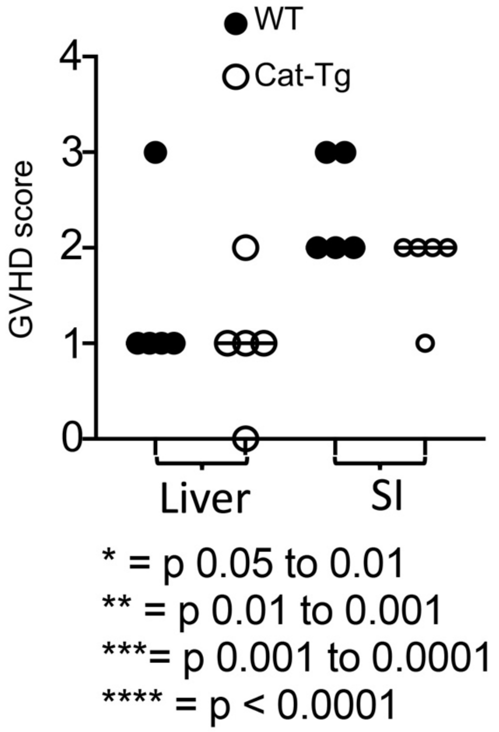

Figure S2. (Related to Figure 8). Quantified scores for GVHD target organ damage. Tissues obtained from recipient mice were graded for GVHD. Quantified GVHD scores for different groups are shown, with median indicated. Statistical analysis was performed using Mann Whitney U test.
